# Comparative cytokine profiling identifies common and unique serum cytokine responses in acute chikungunya and dengue virus infection

**DOI:** 10.1186/s12879-021-06339-6

**Published:** 2021-07-02

**Authors:** Rama Dhenni, Benediktus Yohan, Bachti Alisjahbana, Anton Lucanus, Silvita Fitri Riswari, Dewi Megawati, Sotianingsih Haryanto, Dekrit Gampamole, Rahma F. Hayati, Kartika Sari, Ni Putu Diah Witari, Khin Saw Aye Myint, R. Tedjo Sasmono

**Affiliations:** 1 Eijkman Institute for Molecular Biology, Jakarta, Indonesia; 2 Faculty of Medicine, Universitas Padjadjaran, Bandung, Indonesia; 3 School of Anatomy, Physiology and Human Biology, University of Western Australia, Perth, Australia; 4 Faculty of Medicine and Health Sciences, Warmadewa University, Denpasar, Bali Indonesia; 5 Siloam Hospital, Jambi, Indonesia; 6 Bethesda Hospital, Tomohon, Indonesia

**Keywords:** Chikungunya, Dengue, Cytokine, Host response

## Abstract

**Background:**

Infection by chikungunya (CHIKV) and dengue virus (DENV) can cause a wide spectrum of clinical features, many of which are undifferentiated. Cytokines, which broadly also include chemokines and growth factors, have been shown to play a role in protective immunity as well as DENV and CHIKV pathogenesis. However, differences in cytokine response to both viruses remain poorly understood, especially in patients from countries where both viruses are endemic. Our study is therefore aimed to provide a comparative profiling of cytokine response induced by acute DENV and CHIKV infections in patients with similar disease stages and in experimental in vitro infections.

**Methods:**

By using multiplex immunoassay, we compared host cytokine profiles between acute CHIKV and DENV infections by analysing serum cytokine levels of IL-1α, IL-4, IL-5, IL-8, IL-13, RANTES, MCP-3, eotaxin, PDGF-AB/BB, and FGF-2 from the sera of acute chikungunya and dengue fever patients. We further investigated the cytokine profile responses using experimental in vitro CHIKV and DENV infections of peripheral blood mononuclear cells (PBMCs).

**Results:**

We found that both CHIKV and DENV-infected patients had an upregulated level of IL-8 and IL-4, with the highest IL-4 level observed in DENV-2 infected patients. Higher IL-8 level was also correlated with lower platelet count in dengue patients. IL-13 and MCP-3 downregulation was observed only in chikungunya patients, while conversely PDGF-AB/BB and FGF-2 downregulation was unique in dengue patients. Age-associated differential expression of IL-13, MCP-3, and IL-5 was also observed, while distinct kinetics of IL-4, IL-8, and FGF-2 expression between CHIKV and DENV-infected patients were identified. Furthermore, the unique pattern of IL-8, IL-13 and MCP-3, but not IL-4 expression was also recapitulated using experimental in vitro infection in PBMCs.

**Conclusions:**

Taken together, our study identified common cytokine response profile characterized by upregulation of IL-8 and IL-4 between CHIKV and DENV infection. Downregulation of IL-13 and MCP-3 was identified as a unique cytokine response profile of acute CHIKV infection, while distinct downregulation of PDGF-AB/BB and FGF-2 characterized the response from acute DENV infection. Our study provides an important overview of the host cytokine responses between CHIKV and DENV infection, which is important to further understand the mechanism and pathology of these diseases.

**Supplementary Information:**

The online version contains supplementary material available at 10.1186/s12879-021-06339-6.

## Background

Dengue virus (DENV) is a mosquito-borne single stranded positive-sense RNA virus of the family *Flaviviridae*, genus *Flavivirus*. DENV infection is one of the most important emerging viral infections worldwide, with an estimated 100 million symptomatic infections, resulting in 10,000 deaths annually [1]. Chikungunya virus (CHIKV) is another mosquito-borne single positive-stranded RNA virus of the family *Togaviridae*, genus *Alphavirus*, which also infects millions of people annually, contributing to widespread morbidity and explosive outbreaks of debilitating polyarthralgia [2].

Acute infection by any of the four DENV serotypes or three distinct CHIKV genotypes can cause a wide spectrum of clinical features, many of which are non-specific. Patients infected with either virus can be asymptomatic or present with an undifferentiated febrile illness beginning 3−10 days after infection, with symptoms such as arthralgia, myalgia, headache, rash, and/or nausea [2]. Such similarities can be problematic however, as CHIKV infection is often mistakenly diagnosed as the more common dengue fever, especially in areas where both viruses cocirculate. Chikungunya fever cases have often been found in presumptive dengue cases or during dengue outbreaks when laboratory confirmation was performed [3–5].

Once infected by either virus, the host produces unique innate and adaptive immune responses that result from a complex interplay between virus, host genetics and host immune factors. Cytokines, which broadly also include chemokines and growth factors, are the particular innate immune factors that have been shown to act as inflammatory mediators, playing a key role in both dengue and chikungunya pathogenesis. Previous studies have determined the possible associations between cytokines and either DENV or CHIKV in separate cohorts [6–16]. However, differences in host immune response to both viruses are yet to be compared in vivo nor in vitro. None of these studies has examined CHIKV and DENV cases from Indonesia where both viruses cocirculate [17]. In addition, previous studies have not analysed comparative profiling of cytokine response in acute CHIKV and DENV infections at similar disease stages.

Investigating the immunological profile between these two viral infections is important to understand the mechanism and pathology of the diseases. Development of common immunomodulation treatment strategies can be aided by identifying common immune signature induced by both CHIKV and DENV infection. On the other hand, identifying unique immune response profile of either viruses could lead to biomarker discovery for specific CHIKV or DENV infection. Our study is therefore aimed to compare cytokine response profiles between acute CHIKV and DENV infections in patients from Indonesia as well as in experimental in vitro infections of peripheral blood mononuclear cells (PBMCs) using local clinical virus isolates.

## Methods

### Patient serum samples collection

Archived patient serum samples from cross-sectional prospective studies of dengue and acute febrile illness conducted in nine different cities in Indonesia from 2012 to 2016, including the cities of Jambi, Bandung, Jakarta, Purwokerto, Surabaya, Denpasar, Tomohon, Kendari, and Samarinda were included in this study for analysis of cytokine levels. Some of these studies have been published elsewhere [4, 18–21]. Patients were confirmed positive for either acute CHIKV or DENV infection in the prospective studies by detection of viral RNA using pan-alphavirus/flavivirus RT-PCR and CHIKV-specific real-time RT-PCR [22, 23], or by using the Simplexa Dengue Real-time RT-PCR Kit (DiaSorin, Salugglia, Italy), which can also identify DENV serotypes. The patient serum samples were stored at  -80 °C and uniformly undergone freeze-thaw cycle once before cytokines measurement with multiplex immunoassay.

### Multiplex measurement of serum cytokines

A multiplex immunoassay was performed to measure 10 serum cytokine concentrations using the MILLIPLEX MAP Human Cytokine/Chemokine Magnetic Bead Panel (Millipore, Billerica, MA, USA) according to manufacturer’s instructions. These include: i) pro-inflammatory cytokines: IL-1α and IL-8; ii) anti-inflammatory cytokines: IL-4, IL-5, and IL-13; iii) leukocyte chemoattractant or chemokines: RANTES (also known as CCL5), MCP-3 (also known as CCL7), and eotaxin (also known as CCL11); and iv) growth factors: PDGF-AB/BB and FGF-2.

Each cytokine in the serum samples was captured by cytokine-specific capture antibody-coated magnetic beads, which was further complexed by biotinylated detection antibody and fluorescently labelled streptavidin. Fluorescent signals were detected using the Luminex 100/200 instrument with xPONENT software (Luminex Corp., Austin, TX, USA). Relative fluorescence intensity was converted to cytokine concentration based on standard curves plotted through a 5-parameter logistic curve setting. Calculated minimum detection limit of the kit for IL-1α, IL-4, IL-5, IL-8, IL-13, RANTES, MCP-3, eotaxin, PDGF-AB/BB, and FGF-2 were 9.4, 4.5, 0.5, 0.4, 1.3, 1.2, 3.8, 4.0, 2.2, and 7.6 pg/ml, respectively. For statistical purposes, samples with fluorescence intensity values that could not be interpolated to cytokine concentration based on the standard curve were replaced by half of the minimum detection limit.

### Peripheral blood mononuclear cells (PBMCs) isolation

PBMCs were isolated from the whole blood of four healthy adult donors (median age 33 years) by density gradient centrifugation using Lymphoprep (STEMCELL Technologies). PBMCs were maintained in RPMI 1640 medium supplemented with 2% FBS, 100 U/ml penicillin, 100 μg/ml streptomycin, and 2 mM L-glutamine (Gibco, Carlsbad, CA).

### Viruses

CHIKV strain JMB-192 was isolated from an acute febrile patient in Jambi, Indonesia during a dengue-like outbreak in 2015. Complete genome analysis of this strain has been reported previously, which classified the strain as an Asian genotype [4]. DENV-1 strain JMB-034 and DENV-2 strain JMB-010 were also isolated from dengue patients in Jambi, Indonesia in 2015. Genotyping based on envelope coding sequence classified DENV-1 JMB-034 as genotype I, while DENV-2 JMB-010 as Cosmopolitan genotype [18]. DENV-3 strain SUB-006 and DENV-4 strain SUB-007 were isolated from dengue patients in Surabaya, Indonesia in 2012. Genotyping based on envelope coding sequence classified DENV-3 SUB-006 as genotype I, while DENV-4 SUB-007 as genotype II [19]. The virus was passaged thrice and propagated in Vero (CCL-81) cell culture in MEM medium supplemented with 5% FBS. Virus propagation was done using the multiplicity of infection (MOI) of 0.01 for DENV-1, -2, and -4 and 0.1 for DENV-3 and CHIKV. The generated virus was harvested after significant (more than 80%) cytopathic effect was observed, typically for 5 to 7 days of incubation. Infectious titer was determined by measuring the number of plaque-forming units (PFU) by plaque assay on BHK cell culture as described previously [24]. The stock titer of > 10^6^ PFU/ml was used in the study.

### In vitro infection of PBMCs

Freshly isolated PBMCs were resuspended in complete RPMI at 1 × 10^6^ cells/well in 96-well tissue culture plates and were inoculated separately with four different serotypes of DENV and CHIKV at MOI of 1 in a final volume of 300 μl or mock-infected with medium only. After 2 h incubation at 37 °C with viruses, the inoculum was removed and washed off by centrifugation and fresh medium was added to the cells. At each indicated time point, cell-free supernatant was collected and stored at -80 °C for subsequent analyses of cytokine production by using cytokine-specific sandwich ELISA. Viral RNA load was also measured from PBMC culture supernatant by using real-time quantitative RT-PCR as described previously [23, 25].

### Cytokine-specific ELISA

Cytokine production of infected PBMCs were measured in cell-free supernatants using Human ELISA Ready-Set-Go! kits for four cytokines, i.e. IL-4, IL-8, IL-13, and MCP-3 (all from eBioscience, Affymetrix, Santa Clara, CA, USA) according to manufacturer’ instructions. Calculated minimum detection limit of the kit for IL-4, IL-8, IL-13, and MCP-3 were 2, 2, 4, and 0.3125 pg/ml, respectively.

### Statistical analysis

All statistical analysis was performed using GraphPad Prism version 8. Quantitative data differences between groups were compared by unpaired Student t tests for normally distributed data (based on D’Agostino-Pearson normality test) or by Mann-Whitney tests for non-Gaussian distributed data. For in vitro experiments, a two-way repeated measure ANOVA with Bonferroni multiple comparisons was used to determine significance between groups across all time points. Hierarchical clustering analysis was performed by using ClustVis [26].

## Results

### Characteristics of study population

We selected 32 samples with laboratory-confirmed chikungunya and 43 patients with dengue from our previous cross-sectional prospective studies. All CHIKV-infected patients were at the acute phase of the disease. The dengue group was carefully selected following WHO guidelines and included patients infected with all serotypes: DENV-1 (*N* = 11), DENV-2 (N = 11), DENV-3 (N = 11), and DENV-4 (*N* = 10). We selected dengue patients that were diagnosed as dengue fever (DF, without signs of more severe dengue haemorrhagic fever/DHF or dengue shock syndrome/DSS) because we wanted to compare the cytokine profile with chikungunya patients who shared similar clinical characteristics. Immune related disorders were not ruled out from these selected patients.

All virus strains in chikungunya patient samples were found to be from Asian genotypes based on envelope coding sequences. The selected CHIKV and DENV-infected patients had similar proportion of male/female and fever duration before sample collection (Table 1). However, DENV-infected patients had significantly lower leukocyte and platelet counts compared to CHIKV-infected patients. Furthermore, dengue patients selected in this study were significantly younger than CHIKV-infected patients. Archived healthy adult donor serum samples (*N* = 6) collected from a previous study were included as controls [27].

**Table 1:** Demographic and clinical characteristic of CHIKV and DENV-infected patients

	CHIKV-infected patients (*N* = 32)	DENV-infected patients (*N* = 43)	*P*-value
Sex, male	18/31 (58)	16/32 (50)	0.517
Age in years	30 (1–78), *N* = 31	16 (6–51), *N* = 32	< 0.000
Day of fever in days	3 (1–9), *N* = 22	2 (1–4), *N* = 24	0.494
Leukocyte in 10^3^ cells/μl	6 (3–111), *N* = 22	3 (2–14), *N* = 24	0.001
Platelet in 10^3^ cells/μl	204 (3–309), N = 22	61 (0.2–266), *N* = 32	< 0.000

Data presented as n/N (%) or median (range)

### Serum cytokine comparison revealed differential expression profile between CHIKV and DENV-infected patients as well as between different DENV serotypes

Expression profiles of ten cytokines were analysed and compared between healthy control, CHIKV, DENV, and each DENV serotype groups. These cytokines were selected based on previous studies and were shown to be differentially induced by CHIKV and DENV infection [6–16, 28, 29]. These include: IL-4, IL-5, IL-13, FGF-2, and PDGF-AB/BB that were shown to be upregulated by CHIKV but not (or downregulated) by DENV infection; eotaxin and RANTES that were demonstrated to be upregulated in DENV but not (or downregulated) by CHIKV infection; MCP-3 and IL-8 that were found to be upregulated or unchanged during DENV infection but either downregulated or upregulated in CHIKV infection; as well as IL-1α which was shown to be downregulated in both CHIKV and DENV-infected patients.

**Fig. 1:**
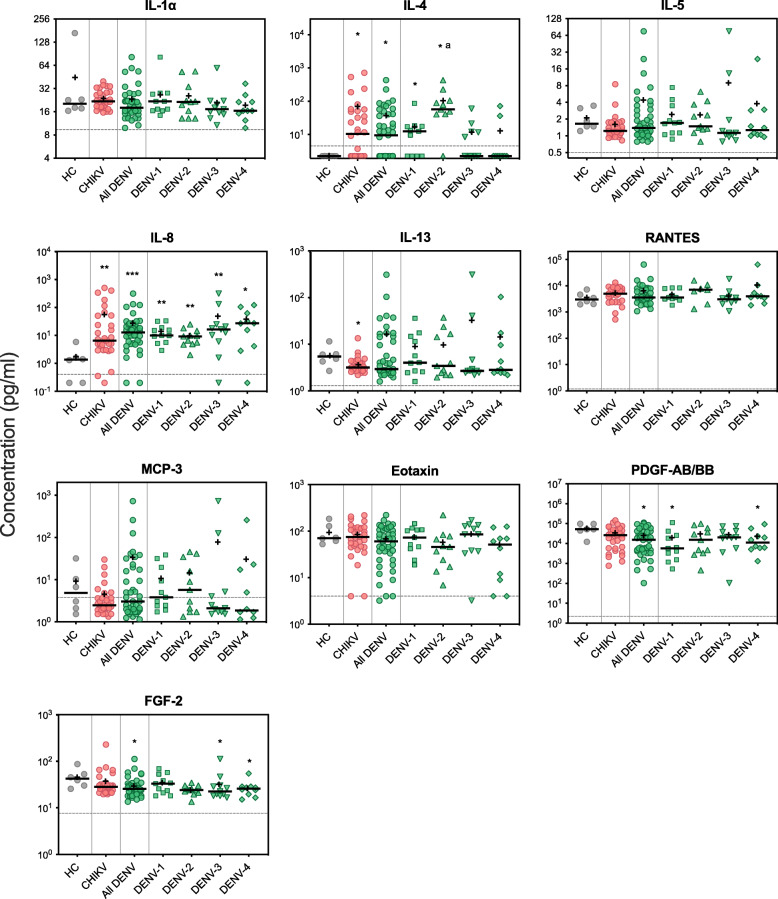
Serum cytokine expression profile of acute CHIKV and DENV-infected patients. Cytokine levels were compared among the groups of 6 healthy controls (HC), 32 CHIKV-infected, and 43 DENV-infected patients, which comprised of 11 DENV-1, 11 DENV-2, 11 DENV-3, and 10 DENV-4 infected patients. Each dot represents the cytokine concentration of an individual. Horizontal bars and plus signs indicate group median and mean, respectively. Assay detection sensitivity limits are indicated by the grey dashed line. Black asterisks represent statistical significance by the Mann-Whitney test between corresponding groups compared to healthy control (*P < 0.05, **P < 0.01). ^a^ Denotes statistical significance between the corresponding groups compared to any other group

Among the cytokines analysed, only IL-4 and IL-8 expression were significantly upregulated in CHIKV and DENV-infected patients compared to the healthy control group. IL-13 expression was significantly downregulated in CHIKV but not DENV-infected patients. Significant downregulation of PDGF-AB/BB and FGF-2 was observed in DENV but not CHIKV-infected patients when compared to healthy control group. No other significant differences were found among expression of IL-1α, IL-5, RANTES, MCP-3, and eotaxin (Fig. 1).

The median level of IL-4 in healthy control group was below detection limit and was five times higher in CHIKV (2.3 pg/ml vs 10.4 pg/ml, *P* = 0.025) and four times higher in DENV (2.3 pg/ml vs 9.5 pg/ml, *P* = 0.036). IL-4 level was not significantly different between CHIKV and all DENV-infected patient group. Interestingly, when DENV group was analysed separately based on the serotype, IL-4 median level in DENV-2 (56.9 pg/ml) was significantly higher compared to all other groups (*P* = 0.011, 0.016, 0.050, 0.019, 0.011, and 0.009 when compared against healthy control, all DENV, CHIKV, DENV-1, DENV-3, and DENV-4, respectively). Such differential expression in DENV-2 was not observed in other cytokines (Fig. 1).

Compared to healthy control, the median level of IL-8 expression was five times higher in CHIKV (1.35 vs 6.53 pg/ml, *P* = 0.006) and nine times higher in DENV-infected patients (1.35 vs 12.7 pg/ml, *P* = 0.000). Although DENV-infected patients had higher IL-8 expression compared to CHIKV, the difference was not statistically significant. Among different DENV serotypes, no significant difference of IL-8 expression was observed (Fig. 1).

IL-13 expression was significantly downregulated in CHIKV compared to healthy control (3.2 vs 5.4 pg/ml, *P* = 0.027). Although the median level of IL-13 in DENV was lower compared to healthy control, the difference was not statistically significant (2.9 vs 5.4 pg/ml, *P* = 0.275) as 32.6% of the DENV patients had IL-13 level above the median of healthy control. Similarly, although MCP-3 expression was not significantly different between healthy control and any DENV groups, 41.9% of DENV patients displayed MCP-3 level above the median level of healthy group (4.9 pg/ml). CHIKV-infected patients had lower median level of MCP-3 expression (2.5 pg/ml) compared to healthy control, although the difference was not statistically significant (*P* = 0.319) (Fig. 1).

**Fig. 2:**
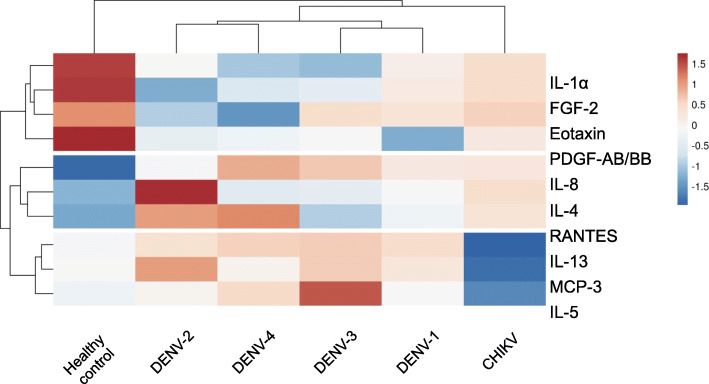
Hierarchical clustering analysis comparing cytokine production of acute CHIKV and DENV-infected patients revealed differential expression profiles between CHIKV and DENV as well as between DENV serotypes. Cytokines are ordered vertically by hierarchical clustering of the rows, which places cytokines with similar expression profiles closer together. Cytokine concentration of patients from the same group are collapsed by taking the mean inside each group and are ordered horizontally by hierarchical clustering so that groups with similar cytokine expression are closer to each other. Rows are centred and unit variance scaling is applied to rows. Therefore, cytokine concentrations are scaled such that a difference of 1 means that the values are one standard deviation away from the average of the row. Red, white, and blue indicates highest, middle, and lowest expression, respectively. Both rows and columns are clustered using Euclidean distance and average linkage

To demonstrate the overall pattern of cytokine expression in the CHIKV and DENV patients, a hierarchical clustering analysis was performed to classify all cytokine responses according to virus groups. The hierarchical clustering analysis identified three clusters of cytokines that had distinct patterns (Fig. 2). The first cluster of cytokines included IL-1α, FGF-2, eotaxin, and PDGF-AB/BB, which were normally expressed in the healthy control, but were reduced in CHIKV and all DENV serotype groups. The second cluster of cytokines included IL-8, IL-4, and RANTES, which were increased in the CHIKV and all DENV serotype groups, while minimally produced in healthy controls. The third cluster of cytokines included IL-13, MCP-3, and IL-5, which had a similar pattern to the second cluster except that expression was markedly reduced in CHIKV-infected patients. Together, these analyses suggested that different expression profiles of cytokines were induced both between CHIKV and DENV infection as well as between different DENV serotypes.

### Serum cytokine levels in acute CHIKV and DENV-infected patients were associated with patient age and platelet count

**Fig. 3:**
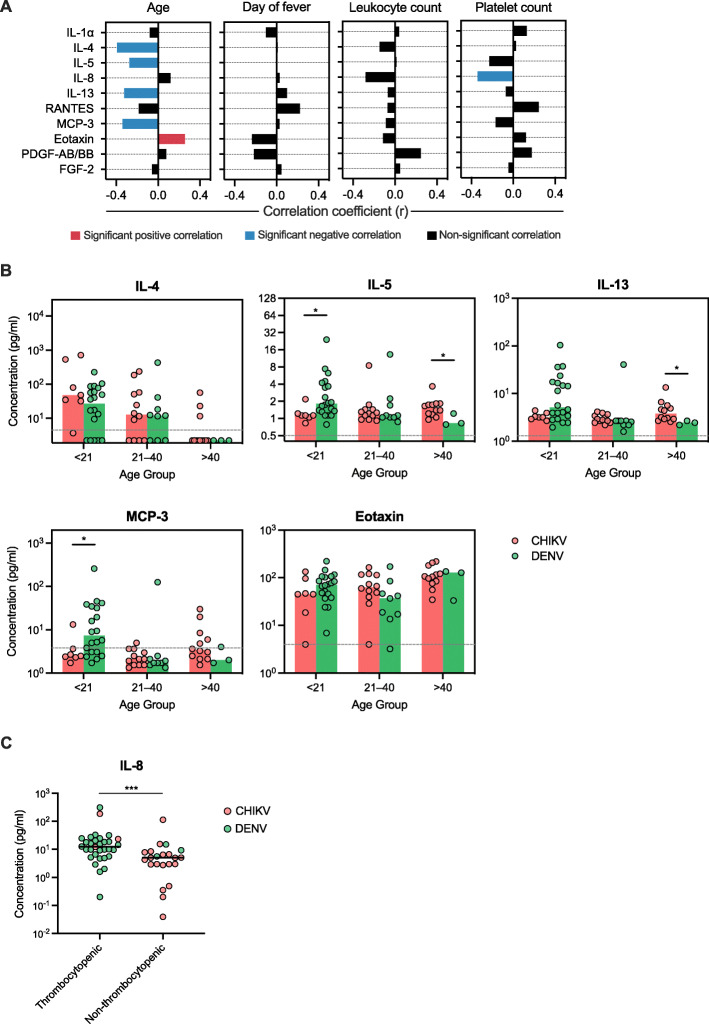
Associations between clinical characteristics and serum cytokine levels of acute CHIKV and DENV-infected patients. (**A**) Spearman analysis was used to test correlations between serum cytokine level and age, duration of fever, leukocyte count, and platelet count. Bars represent correlation coefficient (r) value with colours indicating statistical significance. (**B**) Comparison of IL-4, IL-5, IL-13, MCP-3, and eotaxin serum levels among different age groups between CHIKV and DENV-infected patients. Assay detection sensitivity limits are indicated by the grey dashed line. (**C**) Comparison of IL-8 serum levels between thrombocytopenic (platelet count < 150,000 cells/μl) and non-thrombocytopenic groups. In (**B**) and (**C**), each dot represents the cytokine concentration of an individual. Bars or horizontal lines indicate group median. Black asterisks represent statistical significance by the Mann-Whitney test (*P < 0.05, ***P < 0.001)

We further investigated whether demographic and clinical characteristics of the CHIKV and DENV-infected patients are correlated with serum cytokine level expression. Using Spearman correlation analysis, we found that IL-4, IL-5, IL-13, and MCP-3 serum levels were negatively correlated with patients age (r = − 0.392, *P* = 0.001; r = − 0.273, *P* = 0.030; r = − 0.323, *P* = 0.010; and r = − 0.339, *P* = 0.007; respectively) (Fig. 3A). In contrast, eotaxin serum level was positively correlated with patients age (r = 0.259, *P* = 0.040). Furthermore, increased serum IL-8 level was correlated with lower platelet count (r = − 0.344, *P* = 0.011) (Fig. 3A). No significant sex-based difference in the serum cytokine levels between CHIKV and DENV-infected patients were found. Additionally, no significant correlation was found between serum cytokine levels and day of fever or leukocyte count.

Because age was associated with the level of IL-4, IL-5, IL-13, MCP-3, and eotaxin as mentioned above, we further compared the level of these cytokines between CHIKV and DENV-infected patients stratified according to age group (< 21, 21–40, and > 40 years old) (Fig. 3B). Accordingly, we found that DENV-infected patients of the youngest age group (< 21 years old) had significantly higher serum IL-5 and MCP-3 expression compared to CHIKV-infected patients of the same age group (*P* = 0.011 and 0.027). In contrast, CHIKV-infected patients of the oldest age group (> 40 years old) had significantly higher serum IL-5 and IL-13 expression compared to DENV-infected patients of the same age group (*P* = 0.037 and 0.011) (Fig. 3B).

We also compared IL-8 serum level among patients with thrombocytopenia (platelet count < 150,000 cells/μl) and without thrombocytopenia because Spearman correlation analysis showed that IL-8 serum level was correlated with lower platelet count (Fig. 3A). Unsurprisingly, 90.6% of thrombocytopenic patients were DENV-infected patients, while in contrast 86.4% of the non-thrombocytopenic patients were CHIKV-infected patients. Accordingly, the thrombocytopenic patients had significantly higher level of serum IL-8 compared to the non-thrombocytopenic patients (*P* = 0.000) (Fig. 3C). Comparison of serum IL-8 level between CHIKV and DENV-infected patients within thrombocytopenic and non-thrombocytopenic patients did not show any significant differences (*P* = 0.164 for thrombocytopenic DENV vs CHIKV-infected patients; *P* = 0.086 for non-thrombocytopenic DENV vs CHIKV-infected patients).

### Distinct kinetic of cytokine expression between CHIKV and DENV-infected patients

**Fig. 4:**
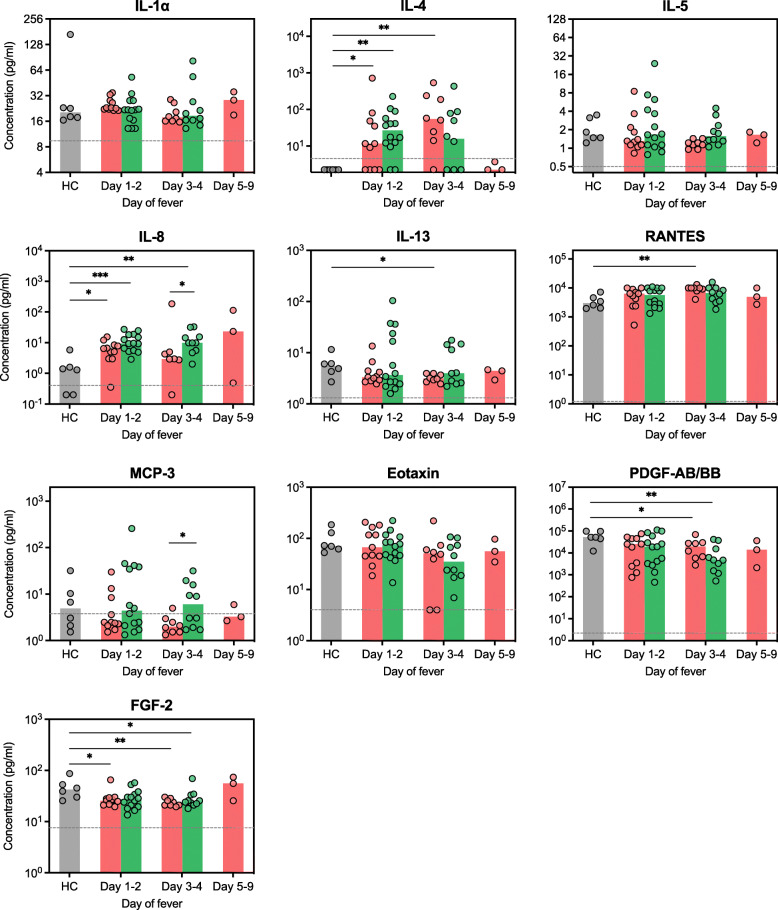
Serum cytokine expression profile in CHIKV and DENV-infected patients on indicated day of fever. Cytokine levels from CHIKV and DENV-infected patients with available day of fever data were segregated into day 1–2, 3–4, and 5–9 and compared with healthy control (HC) group as well as among other groups. Each dot represents the cytokine concentration of an individual. Bars indicate group median. Assay detection sensitivity limits are indicated by the grey dashed line. Black asterisks represent statistical significance by the Mann-Whitney test (*P < 0.05, **P < 0.01, ***P < 0.001)

Although no significant correlation was found between day of fever (day of sampling after symptom onset) and any cytokine level, we nevertheless further analysed the data by segregating the samples into day 1–2, 3–4, and 5–9 comparing CHIKV and DENV-infected patients as well as healthy control to understand the kinetic of cytokine expression. This analysis suggests that IL-4 and IL-8 expressions had distinct kinetic patterns in CHIKV and DENV-infected patients. While both cytokines were significantly upregulated on day 1–2 in both CHIKV and DENV-infected patients (*P* = 0.028 and 0.002, respectively); at day 3–4 IL-4 was significantly upregulated only in CHIKV (*P* = 0.004), but not DENV-infected patients (*P* = 0.090). In contrast, IL-8 was only significantly upregulated in DENV (*P* = 0.002) but not CHIKV-infected patients at day 3–4 (*P* = 0.342) (Fig. 4).

This segregated analysis also supports the findings from pooled analysis including downregulation of IL-13 in CHIKV-patients (*P* = 0.026, day 3–4) as well as significant downregulation of PDGF-AB/BB (*P* = 0.003) and FGF-2 (*P* = 0.021) on day 3–4 in DENV-infected patients when compared to the healthy control. Interestingly, expression of FGF-2 in CHIKV-infected patients also showed a decreasing trend with significant difference from healthy control on day 1–2 (*P* = 0.019) and 3–4 (*P* = 0.004). There was also an increasing trend of RANTES expression in CHIKV-infected patients with significant difference on day 3–4 compared to healthy control (*P* = 0.003) (Fig. 4). Unfortunately, the limited number of samples with available day of fever data did not allow us to make a statistically meaningful comparison between DENV serotypes. Overall, this analysis suggests distinct kinetic of cytokine expression profile in CHIKV and DENV-infected patients.

### In vitro CHIKV and DENV infection of PBMCs induce expression of IL-8, IL-13, and MCP-3, but not IL-4

We further performed in vitro experiments using PBMCs from healthy donors to measure the cytokine production after infection with CHIKV, DENV-1, 2, 3, and 4, which allowed us to characterize the kinetics of cytokine induction during CHIKV and DENV infection under controlled experimental settings. We used PBMCs, which are known to support CHIKV and DENV replication and have been used in a number of studies investigating host immune response [30–34]. Measurement of viral RNA level from PBMC culture supernatant showed that there was an increase of viral RNA between 6- and 72-h post infection suggesting that the PBMCs were infected with the viral strain we used (see Additional file 1). We focused on IL-4 and IL-8, which were significantly upregulated in both CHIKV and DENV-infected patient samples, as well as IL-13 and MCP-3, which were found to be upregulated in 32.6–41.9% of the DENV-infected patients and conversely downregulated in CHIKV-infected patients. IL-13 and MCP-3 serum levels were also found to be age-dependent (Fig. 3A and B).

**Fig. 5:**
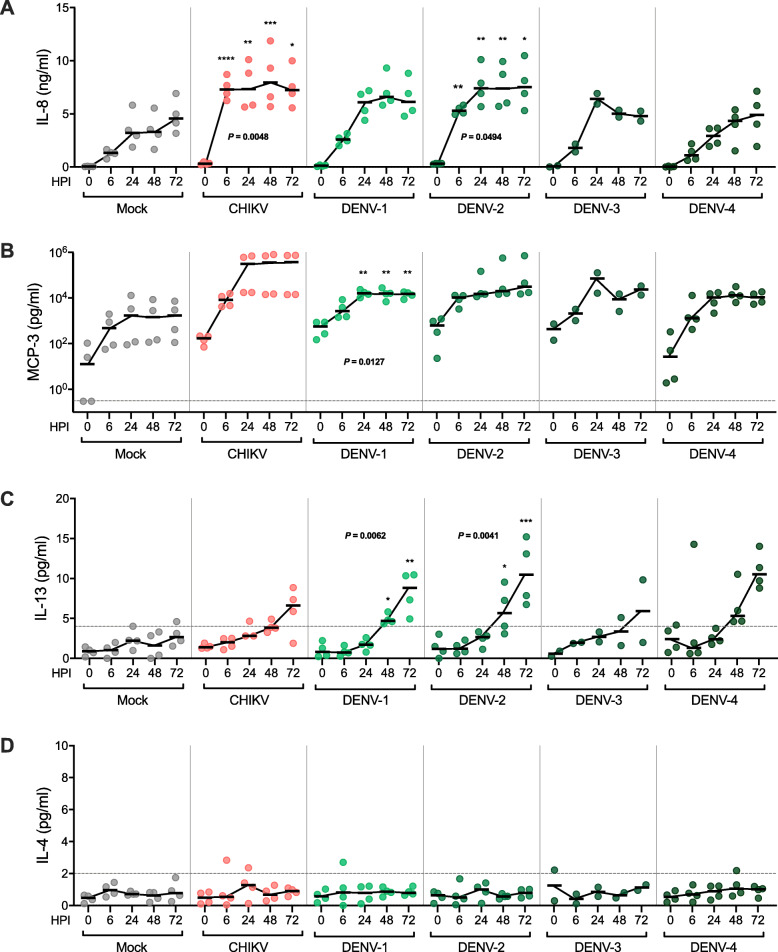
In vitro CHIKV and DENV infection of PBMCs induced expression of IL-8, IL-13, and MCP-3, but not IL-4. PBMCs from healthy donors were isolated and infected with CHIKV, DENV-1, -2, -3, -4 (MOI if 1) or mock-infected, and supernatant was collected at 0, 6, 24, 48, and 72 h post infection (HPI). Levels of (**A**) IL-8, (**B**) MCP-3, (**C**) IL-13, and (**D**) IL-4 in supernatant were measured by cytokine-specific sandwich ELISA. Each dot represents the cytokine concentration of an individual donor. Horizontal black bars indicate the respective group median, which are connected with the continuous black line. ELISA kit detection sensitivities are indicated by the grey dashed line. Statistical significances of time and treatment effects comparing either CHIKV, DENV-1, -2, -3, or-4 infected with mock infected PBMCS were analysed by the two-way repeated measure ANOVA. *P* values from statistically significant results are shown, while black asterisks represent statistical significance via Bonferroni corrected post hoc test (*P < 0.05, **P < 0.01, ***P < 0.001, ****P < 0.0001)

CHIKV infection of PBMCs induced significant production of IL-8 compared with mock infection (*P* = 0.005, multiple time point comparison) (Fig. 5A). IL-8 production was already evident 6 h post CHIKV infection and stably maintained for up to 72 h post infection. Similarly, DENV-2 infection also induced significant production of IL-8 compared to mock infected PBMCs (*P* = 0.049, multiple time point comparison); the median level across all time points was not significantly different when compared to that infected with CHIKV (not shown). In contrast, DENV-1, DENV-3, and DENV-4 infection induced minimal production of IL-8 and the median levels throughout the infection were not statistically significant (Fig. 5A).

Significant production of MCP-3 was only observed in DENV-1 infected PBMCs (*P* = 0.013, multiple time point comparison), which peaked at 24 h post infection compared to mock infected PBMCs (Fig. 5B). Unlike DENV-1 infection, CHIKV, DENV-2, DENV-3, and DENV-4 infection of PBMCs minimally induced MCP-3 secretion which were not statistically significant.

Marked increase of IL-13 expression was observed in DENV-1 infected PBMCs compared to mock infection (*P* = 0.006, multiple time points comparison); IL-13 production was above the limit of detection at very late time points (i.e. > 48 h post infection) (Fig. 5C). This trend was also observed in DENV-2 infected PBMCs (*P* = 0.004, multiple time points comparison). Comparison between DENV-1 and DENV-2-induced IL-13 expression across all time points did not reveal significant differences. CHIKV, DENV-3, and DENV-4 infection did not induce statistically significant IL-13 production.

IL-4, although highly expressed in the CHIKV and DENV-infected patients, was not detected in our in vitro experiments (Fig. 5D). Infection of PBMCS with either CHIKV, DENV-1, -2, -3, and -4 did not produce detectable levels of IL-4, suggesting that PBMCs may not be the major source of IL-4 in the CHIKV and DENV-infected patients.

## Discussion

Human immune responses induced by CHIKV and DENV infections are complex and characterized by robust cellular and humoral immune response together with high levels of circulating cytokines and chemokines. Both CHIKV and DENV are endemic in tropical countries including Indonesia and may cause acute fever with a wide spectrum of clinical features in patients. Understanding the dynamics of cytokine response in each of these diseases is therefore fundamental to identifying the potential mechanisms associated with pathogenesis and protective immunity. In this study, we provide a comparative profiling of cytokine response induced by acute DENV and CHIKV infections in patients and in vitro, which revealed both common and differential cytokine expression profiles.

Notably, our study found that IL-8, a pro-inflammatory cytokine, was commonly upregulated in both CHIKV and DENV-infected patients. This was also evident in in vitro experiments although DENV-1, DENV-3 and DENV-4 infected PBMCs produced less IL-8 compared to DENV-2. Interestingly, higher IL-8 level was found to be correlated with lower platelet count in patients, predominantly in those with DENV infection, as observed in earlier studies [7, 10, 35]. This suggests that IL-8 might be involved in the pathophysiology of thrombocytopenia in dengue.

Similar to IL-8, IL-4 was markedly upregulated in both CHIKV and DENV-infected patients. Interestingly, DENV-2 patients exhibited the highest levels of IL-4 compared to those with CHIKV and other DENV serotypes. Some studies have reported the upregulation of serum IL-4 in CHIKV [13, 14] and DENV-infected patients [7], as well as increased number of IL-4 expressing PBMC of paediatric dengue haemorrhagic fever [36], although the differential expression of IL-4 by DENV-2 (i.e. markedly upregulated in DENV-2, but not/only slightly in other serotypes) has not been reported before. Despite its prominent role in allergy and immunity to parasitic infection, IL-4 also has known importance in inducing B cell response and humoral immunity [37]. Whether prominent IL-4 expression in DENV-2 infection is a biomarker associated with this serotype warrants further investigation.

IL-13, which shares a receptor and signalling pathway with IL-4, was markedly downregulated in CHIKV but not in any of the DENV-infected patients. This result was somewhat supported by our in vitro experiments, in which IL-13 induction was significantly observed in DENV-1 and DENV-2 but not in CHIKV-infected PBMCs. Studies in experimental mouse models have shown that IL-13 and IL-4 regulate different antibody class expression via IFN-γ-dependent signalling by negatively regulating subclass IgG1 and IgG2a switching, respectively [38]. Interestingly, among IgG class, IgG1 antibodies were the predominant isotype produced during acute DENV infection [39], while IgG3 antibodies were the predominant isotype expressed during acute CHIKV infection [40]. This suggests a possible role of these cytokines in regulating differential antibody isotype expression in acute CHIKV and DENV infection.

Studies regarding IL-13 expression in CHIKV and DENV infection have been limited. However, one study has reported increased IL-13 in severe but not mild dengue [7], while another study found a moderately increased level in acute CHIKV infection [14, 41]. These discrepancies to our study could be explained by disease severity and strain of the infecting viruses; our study included only patients with DF without signs of more severe DHF or DSS, and all of our chikungunya patients were infected with the Asian genotype and not the Central African lineage which might induce distinct innate immune response [42]. It is also worth noting that hierarchical clustering analysis also found that MCP-3 and IL-5 have similar patterns of expression with IL-13. Interestingly, MCP-3 and IL-5 levels were significantly upregulated in DENV-infected patients only in the youngest age group, suggesting age-associated regulation of these cytokines in acute DENV-infections.

In contrast to IL-13, the level of growth factors PDGF-AB/BB and FGF-2 was significantly downregulated in DENV but not in CHIKV-infected patients. Other studies have also observed downregulation of PDGF and FGF-2 in dengue patients [6, 11], while FGF-2 was reported to be upregulated in chikungunya patients [15, 43]. Downregulation of these cytokines may reflect a physiological response to tissue damage caused by DENV, but not by CHIKV infection. Interestingly, Zika virus (ZIKV)-infected patients have also been found to have lower level of PDGF-BB compared to healthy controls [44], suggesting a common physiological response between DENV and ZIKV infection.

Our study has several potential limitations. First, the sample size was limited, especially for the healthy control group. Second, demographic and clinical data from some cases were missing which prevent us to do meaningful statistical comparison between subgroups. Third, we only analysed serum cytokines from a single time point during the acute phase of the diseases. Investigating serum cytokine concentration at earlier and/or later time points to study cytokine kinetic profiles throughout the phases of illness might revealed additional findings. Fourth, we only measured ten cytokines. Many other cytokines not included in our study may have significant role in these diseases. Fifth, we used only one representative strain for CHIKV and for each DENV serotype in our in vitro study; hence, the differences observed in cytokine production between the viruses may not apply generally.

## Conclusions

In summary, we identified cytokine response profiles common between acute CHIKV and DENV-infected patients characterized by upregulation of IL-8 and IL-4, with highest IL-4 level observed in DENV-2 infected patients. We also identified a unique cytokine response profile of acute CHIKV infection, characterized by downregulation IL-13 and MCP-3 as well as distinct downregulation of PDGF-AB/BB and FGF-2, which distinguished the response from acute DENV infection. We believe that our study provides an important overview of the cytokine response between CHIKV and DENV infection, which may be critical to identify host immune responses associated with protective immunity and pathogenesis of these diseases.

## Supplementary Information

**Additional file 1.** Viral load kinetics of CHIKV, DENV-1, DENV-2, DENV-3, and DENV-4 infection on healthy donor-derived PBMC culture. PBMC culture supernatants were analysed for viral RNA load by using real-time quantitative RT-PCR at indicated time points. Data are expressed as mean ± SEM of viral RNA plaque-forming unit equivalent (PFUeq)/ml.

## Data Availability

The datasets used and/or analysed during the current study are available from the corresponding author on reasonable request.

## Abbreviations

CHIKV: Chikungunya virus
DENV: Dengue virus
DF: Dengue fever
DHF: Dengue haemorrhagic fever
DSS: Dengue shock syndrome
HC: Healthy controls
PFU: Plaque-forming unit
PBMCs: Peripheral blood mononuclear cells
ZIKV: Zika virus
