# Supplementary figures and images for: Comparative cytokine profiling identifies common and unique serum cytokine responses in acute chikungunya and dengue virus infection
Source: BMC Infect Dis. 2021 Jul 2;21:639. doi: 10.1186/s12879-021-06339-6 (PMC8254284; doi:10.1186/s12879-021-06339-6)

**DENV-1**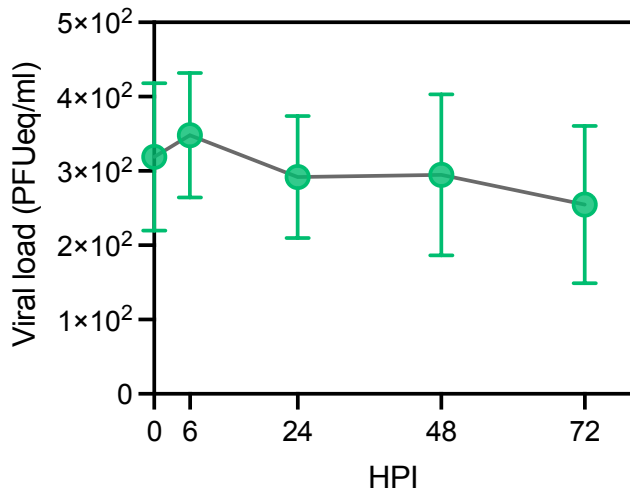**DENV-2**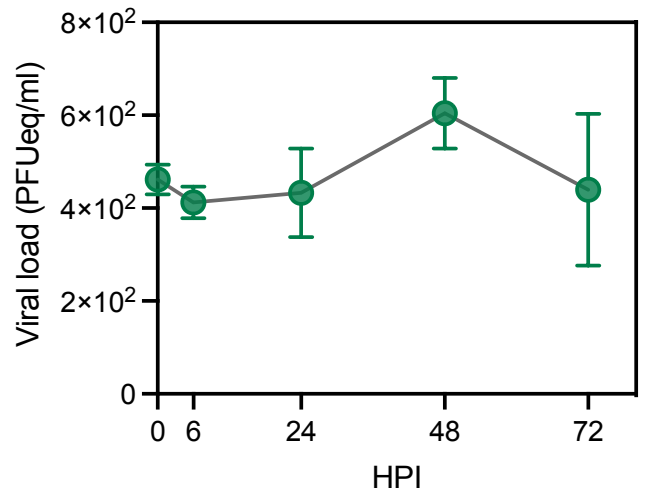**DENV-3**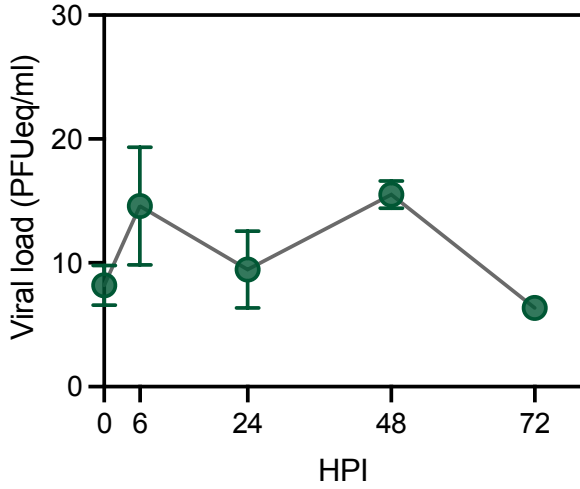**DENV-4**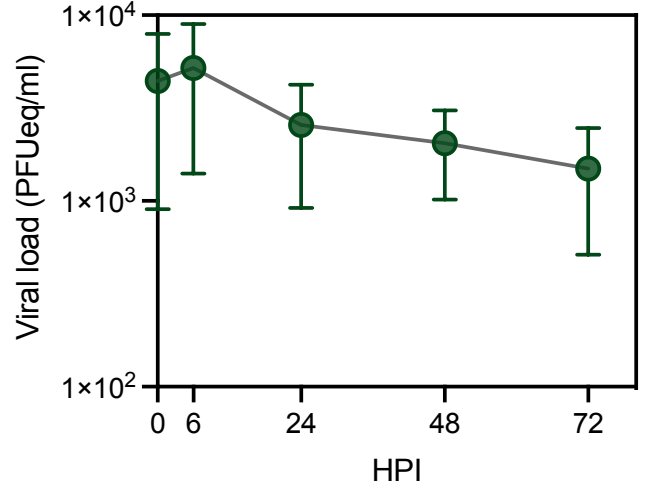**CHIKV**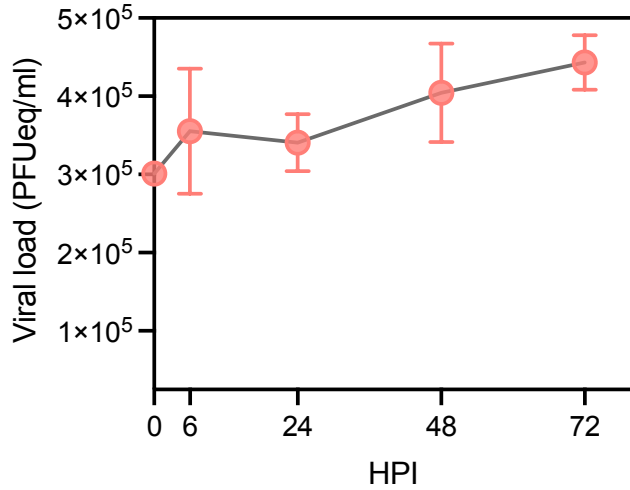

Supplement: Supplementary file 1 — Additional file 1. Viral load kinetics of CHIKV, DENV-1, DENV-2, DENV-3, and DENV-4 infection on healthy donor-derived PBMC culture. PBMC culture supernatants were analysed for viral RNA load by using real-time quantitative RT-PCR at indicated time points. Data are expressed as mean ± SEM of viral RNA plaque-forming unit equivalent (PFUeq)/ml. [file 12879_2021_6339_MOESM1_ESM.pdf]
